# Comparative Evaluation of Microbial Adhesion on Provisional Crowns Fabricated With Milled Polymethyl Methacrylate (PMMA) and Conventional Acrylic Resin: A Prospective Clinical Trial

**DOI:** 10.7759/cureus.64469

**Published:** 2024-07-13

**Authors:** Pooja Singh, Amrutha Shenoy, Deepak Nallaswamy, Subhabrata Maiti

**Affiliations:** 1 Department of Prosthodontics, Saveetha Dental College and Hospital, Saveetha Institute of Medical and Technical Sciences, Saveetha University, Chennai, IND

**Keywords:** single crowns, cfu, s. mutans, provisional crowns, pmma

## Abstract

Introduction

Provisional prosthetic restorations play a crucial role in dentistry by protecting dentinal tubules, offering thermal insulation, and ensuring a precise fit during dental treatments. Computer-aided design and computer-aided manufacturing (CAD/CAM) have improved polymethyl methacrylate (PMMA), enhancing its mechanical properties such as hardness and resistance compared to traditional methods. However, bacterial accumulation remains a challenge due to inherent surface roughness. This study aims to assess and compare *Streptococcus mutans* adhesion on milled PMMA and conventional self-cure acrylic resin, providing insights into their microbial interaction dynamics.

Materials and methods

This study was a prospective trial approved by the Institutional Human Ethical Committee (SRB-IHEC) (registration number: IHEC/SDC/PROSTHO-2104/24/045) and registered in the Clinical Trial Registry, India (registration number: CTRI/2024/05/068196). The study involved 20 patients requiring single crowns in the right and left molar regions. Two groups were established: Group I (the milled PMMA group) and Group II (the conventional PMMA group). Criteria for participant selection and exclusion were set. A total of 120 swab samples from the buccal mucosa and tooth surfaces were collected before tooth preparation (the baseline) at one week and three weeks. Culture for *S. mutans* was done, and colony-forming units were counted. Data analysis was carried out using IBM SPSS Statistics for Windows, Version 26 (Released 2019; IBM Corp., Armonk, New York, United States). An independent sample t-test was employed to compare the two materials for crowns. To analyze changes over time within each group, a repeated-measures analysis of variance (ANOVA) was conducted. When the ANOVA test indicated significance, Tukey’s post-hoc test was utilized for pairwise mean comparison. The level of significance was set at P < 0.05.

Results

The mean colony-forming units (CFU) counts for the milled PMMA group were 4.46 ± 0.167 CFU at baseline, 4.163 ± 0.058 CFU at one week, and 3.87 ± 0.19 CFU at three weeks. The mean CFU counts for the conventional PMMA group were 4.41 ± 0.13 CFU at baseline, 4.29 ± 0.114 CFU at one week, and 4.16 ± 0.108 CFU at three weeks. At baseline (before cementation), there was no difference between milled PMMA and conventional PMMA (P = 0.578). After one week, a significant difference between milled PMMA and conventional PMMA was observed (P < 0.005). After three weeks, a significant difference between milled PMMA and conventional PMMA persisted (P < 0.005).

Conclusion

There was a significant reduction in microbial adhesion in both the milled and conventional PMMA groups. However, milled PMMA demonstrated a greater decrease in microbial adhesion as compared to conventional PMMA.

## Introduction

Provisional prosthetic restorations protect dentinal tubules from leakage and safeguard prepared dental tissues until permanent prostheses are ready. These materials are vital in prosthetic treatments, providing thermal insulation and ensuring a precise fit with dental tissue [1]. In addition to their protective role, they have diagnostic and pre-prosthetic roles, such as correcting occlusal plane irregularities, enhancing vertical dimension, and shaping or maintaining gingival position [2]. One of the most commonly used provisional materials is polymethyl methacrylate (PMMA), which is available as a fine powder made by blending polymerized methyl methacrylate with a liquid monomer [3].

Since its inception in the 1980s, digital dentistry has significantly transformed various aspects of dental practice [4,5]. Initially, the focus was on developing computer-aided design/computer-aided manufacturing (CAD/CAM) systems for creating complete removable prostheses. In recent years, CAD/CAM technology has expanded to produce a wide range of prosthetic restorations, including implants and full dentures [6]. Although CAD/CAM PMMA shares similar chemical properties with conventionally heat-cured PMMA, it surpasses it in hardness, flexural strength, and impact strength [7]. These improved mechanical properties make milled PMMA suitable for long-term temporary restorations, lasting up to one year [8,9]. Furthermore, the increased hydrophobicity of CAD/CAM PMMA compared to its conventional counterpart reduces plaque accumulation on these prostheses [10].

Provisional restorations worn for extended periods can lead to significant bacterial colonization on their surfaces, which critically affects the success of the restoration. Due to the increased surface roughness and poor marginal fit of provisional restorative materials, bacterial colonization is more pronounced compared to permanent restorative materials [11]. Various surface characteristics, such as roughness, influence both the quantity and quality of bacterial accumulation [12]. Increased surface roughness enhances microbial adhesion, making it challenging to remove bacteria from pits and grooves [13]. *Streptococci*, commonly known as "early colonizing bacteria," are frequently found on these surfaces and play a primary role in the development of tooth caries [14].

Various studies have investigated bacterial accumulation on permanent prosthodontic materials [15]. Data on microbial adhesion to prosthodontic provisional materials is limited. Therefore, this study aims to compare the surface adhesion of *Streptococcus mutans* on milled PMMA versus conventional cold-cure acrylic resin at different time intervals.

## Materials and methods

The study was designed as a prospective, single-center, double-blinded, split-mouth, randomized controlled trial conducted at Saveetha Dental College in Chennai, India. It was approved by the Institutional Human Ethical Committee (SRB-IHEC) (registration number: IHEC/SDC/PROSTHO-2104/24/045) and registered in the Clinical Trial Registry, India (registration number: CTRI/2024/05/068196). The study enlisted 20 patients necessitating single crowns in the right and left first molars, and the sample size was decided using the G power calculation (G*power 3.0.10) [16]. A meticulous case history involving extraoral examination, intraoral examination, and evaluation of temporomandibular joints and associated structures, accompanied by patient-informed consent, was conducted before commencing the study.

Participants

**Figure 1:**
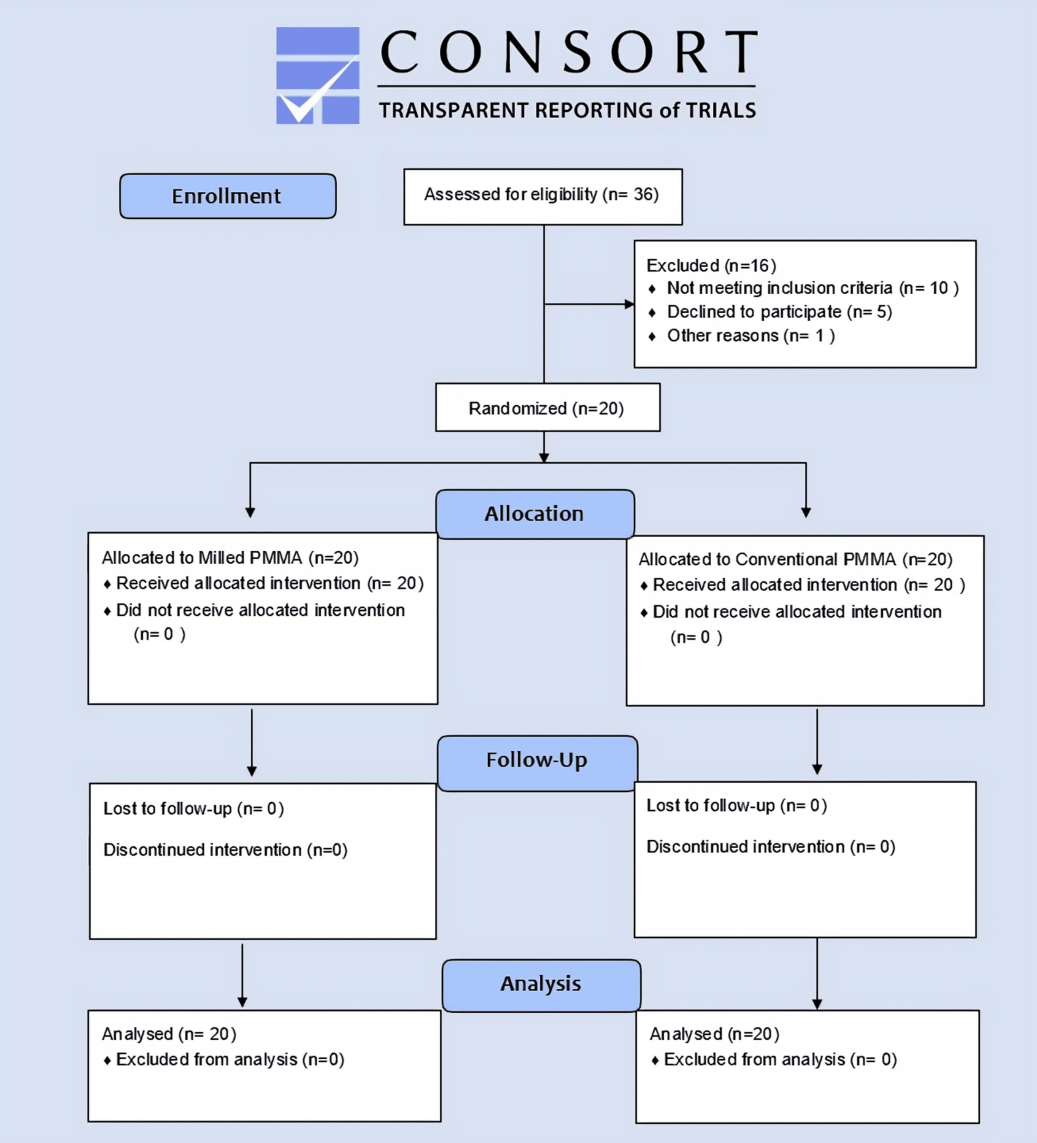
CONSORT flowchart describing patient enrollment, randomization, allocation, follow-up, and analysis CONSORT: Consolidated Standards of Reporting Trials; PMMA: polymethyl methacrylate

This study focused on patients who needed single crowns in the right and left first molars (Figure 1). Randomization was done using a computer-generated sequence of random numbers. Sequences are sealed in opaque envelopes to ensure sufficient concealment. The inclusion criteria for this study include individuals between the ages of 20 and 35 who are systemically healthy and in need of single crowns. Additionally, participants should have a gingival index of 0, a plaque index score of 0, no periodontal pockets, and negative bleeding on probing [17]. On the other hand, the exclusion criteria encompass periodontally compromised teeth, individuals who are pregnant or lactating, those with a history of systemic diseases or other debilitating conditions, individuals on steroids, anticoagulants, aspirin, or other drugs, patients with harmful oral habits, and those unwilling to give consent for the study.

Intervention 

Tooth preparation was done following all the principles of tooth preparation, and impressions were made using a single tray impression technique with the addition of silicone impression material (Zhermack SpA Elite HD+, Italy). Casts were poured with type IV die stone (Zhermack Stone, Italy). A total of 120 swab samples were collected from patients and split into two groups based on the material of the provisional restoration.

Group I (milled PMMA crowns) had PMMA temps designed using 3 Shape designing software (3 Shape, Copenhagen, Denmark) and milled with the help of a 5-axis milling machine (IMES iCore, CORiTEC 350i milling machine®) with the multilayered PMMA blank. 

Group II (conventional PMMA crowns) had conventional cold-cure acrylic provisional restorations (DPI self-cure tooth molding powder, Bombay Bumrah Trading Corp., Mumbai, India) made using the indirect technique with the help of manual wax up on the primary cast. 

**Figure 2:**
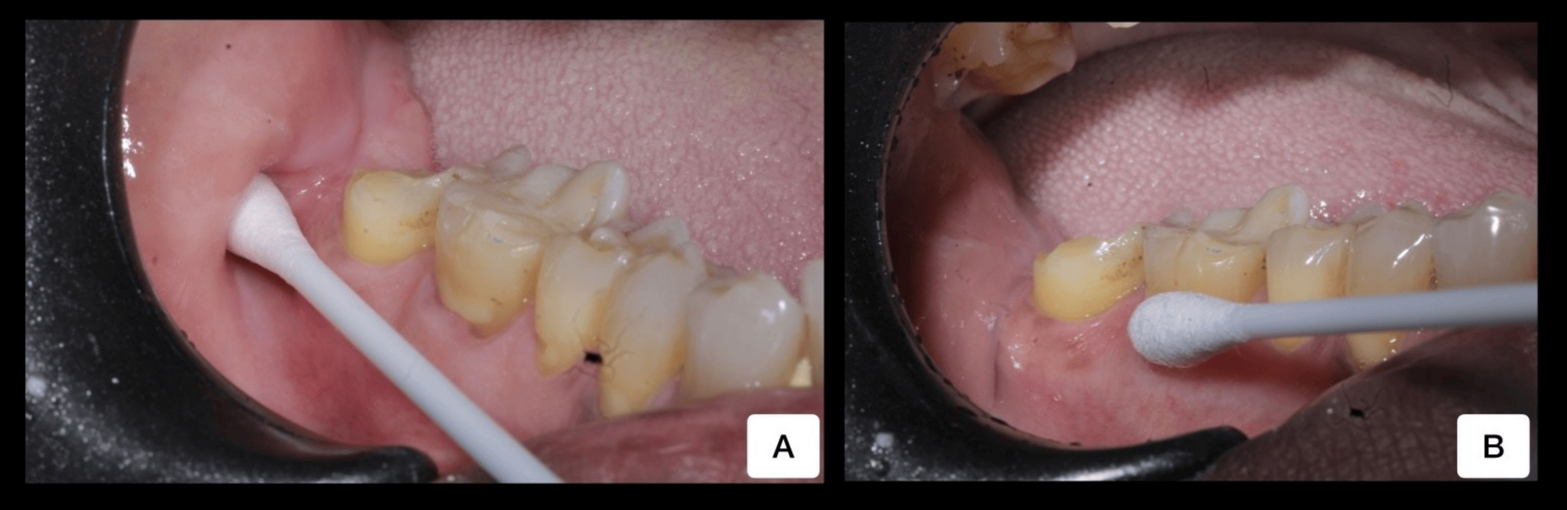
Swab samples collected from the patient’s mouth A: buccal mucosa; B: tooth surface

The crowns were then cemented using temporary cement (3M, RelyX Temp, 3M ESPE, Maplewood, Minnesota, USA). Swabs from buccal mucosa and crown surfaces on both sides in the timeline of baseline, one week, and three weeks were taken from each patient (Figure 2). 

Microbial cultures

Sterile swabs were used to collect samples from the oral cavity of participants, and these swabs were then suspended in sterile saline. Brain-heart infusion broth was used to culture *S. mutans* for colony-forming units (CFU) determination. Of the sample suspension, 100 µL was inoculated onto agar plates and spread evenly. The culture plates were then incubated at 37°C for 48 hours in a microaerophilic environment. After incubation, colonies of *S. mutans* were counted to determine the CFU per sample, with each colony representing a single cell or group of cells. CFU counts were recorded and analyzed statistically to compare samples and assess any associations.

Statistical analysis

Data was collected and tabulated using Microsoft Excel software, Version 16.78.3, 2023 (Microsoft Corporation, Redmond, Washington, United States). The Kolmogorov-Smirnov normality test was applied to evaluate the data distribution. Results were expressed as mean and standard deviation (SD) values. To normalize the data due to the wide range of *S. mutans* counts, a logarithmic transformation (log 8) was performed on each CFU value before statistical evaluation. An independent sample t-test compared the two materials for crowns, while an analysis of variance (ANOVA) examined the changes over time within each group. Tukey’s post-hoc test was conducted for pairwise comparisons between means. Data analysis was carried out using IBM SPSS Statistics for Windows, Version 26 (Released 2019; IBM Corp., Armonk, New York, United States). The level of significance was set at P < 0.05.

## Results

*S. mutans* count: comparison of the two crowns

In this study, 120 samples were tested to compare *S. mutans* counts between milled PMMA crowns (Group I) and conventional acrylic resin crowns (Group II). At baseline, no significant difference was found in the bacterial counts between the two groups (P = 0.578), indicating similar initial conditions. However, after one week, a significant difference emerged, with the milled PMMA crowns showing substantially lower *S. mutans* counts compared to the conventional PMMA crowns (P < 0.005). This trend continued at the three-week mark, where the milled PMMA crowns maintained significantly lower bacterial counts (P < 0.005). This early and sustained reduction in bacterial counts suggests that the milled PMMA material may be more effective in controlling bacterial growth shortly after application (Table 1).

**Table 1:** Comparison of mean colony-forming units (CFU) values between the groups at different time intervals *Significant at P < 0.05, p-value was derived using independent sample t-test. PMMA: polymethyl methacrylate

Timeline	Groups	N	Mean ± SD	Std. Error	95% CI		t	df	P-value
					Lower	Upper			
Baseline	Milled PMMA	20	4.46± 0.167	0.0037	-0.047	0.145	1.033	38	0.578
										Conventional PMMA	20	4.41 ± 0.13	0.029
	1 week	Milled PMMA	20	4.163 ± 0.058						0.129	-0.185	-0.070	-4.46	38	0.000*
Conventional PMMA					20	4.291 ± 0.114	0.026								
3 weeks		Milled PMMA	20	3.87 ± 0.19	0.048	-0.409	-0.19	-5.537	38	0.000*					
	Conventional PMMA										20	4.16 ± 0.108	0.108

Change by time with individual materials

Milled PMMA Crowns

The mean CFU counts for the milled PMMA group were 4.46 ± 0.167 at baseline, 4.163 ± 0.058 at one week, and 3.87 ± 0.19 at three weeks. Within this group, a significant reduction in *S. mutans* counts was observed over the study period. At baseline, the mean CFU count was 4.46 ± 0.167. After one week, this count significantly decreased to 4.163 ± 0.058 (P < 0.005). The reduction continued over the next two weeks, with the mean CFU count dropping further to 3.87 ± 0.19 at the three-week mark (P < 0.005) (Table 2). The consistent decrease in bacterial counts highlights the efficacy of milled PMMA crowns in reducing microbial presence shortly after placement. The rapid decline in *S. mutans* suggests that milled PMMA crowns may provide an effective barrier against bacterial colonization.

**Table 2:** Comparison of mean colony-forming unit (CFU) values within the groups at baseline, one week, and three weeks *Significant at P < 0.05, the p-value was derived using the Tukey post-hoc test. PMMA: polymethyl methacrylate

Groups	Timeline	Mean Difference	Std. Error	95% Confidence Interval (CI)		P-value
				Lower	Upper	
Milled PMMA	Baseline vs. 1 week	0.295	0.047	0.180	0.409	0.000*
							Baseline vs. 3 weeks	0.587	0.047	0.473	0.702	0.000*
	1 week vs. 3 weeks	0.292	0.047	0.178	0.407	0.000*						
	Conventional PMMA	Baseline vs 1 week	0.118	0.037	0.029	0.208	0.007
								Baseline vs 3 weeks	0.251	0.037	0.161	0.341	0.000*
1 week vs 3 weeks		0.133	0.037	0.043	0.222	0.002*							

Conventional PMMA Crowns

The mean CFU counts for the conventional PMMA group were 4.41 ± 0.13 at baseline, 4.29 ± 0.114 at one week, and 4.16 ± 0.108 at three weeks. For this group, the reduction in *S. mutans *counts was more gradual. At baseline, the mean CFU count was 4.41 ± 0.13. After one week, the decrease in bacterial counts was not statistically significant, with the mean CFU count slightly lowering to 4.29 ± 0.114 (P > 0.005). However, by the three-week mark, a significant reduction was observed, with the mean CFU count dropping to 4.16 ± 0.108 (P < 0.005) (Table 2). This indicates that while conventional PMMA crowns do eventually reduce bacterial counts, they do so at a slower rate compared to milled PMMA crowns. The delayed reduction in *S. mutans* suggests that conventional PMMA crowns may be less effective in the immediate post-placement period, potentially allowing for a higher risk of bacterial proliferation and associated complications during the initial weeks after crown placement.

## Discussion

The clinical trial aimed to assess the surface adhesion of *Streptococcus mutans* on milled PMMA compared to conventional cold-cure acrylic resin. Throughout the study, a significant decrease in the CFU of *S. mutans* was observed in both groups. The reduction in the milled PMMA group was more pronounced than in the conventional PMMA group, leading to the rejection of the null hypothesis.

One of the most significant factors influencing microbial adhesion is the composition of the materials, which plays a significant role in surface characteristics, thereby affecting bacterial adherence. The physical and mechanical properties of materials may favor bacterial colonization [18]. Studies in the literature have concluded that the presence of fillers and monomers increases the adhesion of several bacteria on certain dental materials [19,20]. The potential harmful effects of PMMA materials on bacterial cells might explain the reduced adhesion observed in these groups. Additionally, the chemical byproducts and heat released during polymerization may impact microbial cell viability. 

Al-Dwairi et al. stated that milled PMMA has superiority in physical properties like wettability, surface roughness, and surface hardness as compared to conventional PMMA [21]. Another study done by Srinivasan et al. showed similar results in terms of the mechanical properties of milled PMMA. These findings could explain the reduced bacterial accumulation in the milled PMMA group compared to the conventional acrylic resin group [22].

Most provisional crown bridges are made chairside using an over-impression method with resin-based temporary crown materials. Since the provisional prosthesis needs to remain in place for a few weeks, the materials and fabrication techniques play a crucial role. Chairside manipulation of provisional restorations with PMMA-based materials has drawbacks, such as poor mechanical strength, surface texture, and precise fit. In contrast, CAD/CAM PMMA blocks are made in optimal industrial manufacturing conditions, providing better physical properties than manually fabricated restorations [23]. Consequently, CAD/CAM PMMA exhibits superior qualities in terms of bacterial colonization and surface characteristics compared to conventional PMMA. This observation aligns with previous reports indicating that milled provisional restorations have increased mechanical strength compared to directly fabricated temporary restorations using PMMA-based materials [24]. Additionally, the better clinical fit of milled provisional crowns reduces the risk of bacterial adhesion to the surface [25,26].

Our study has a few limitations. Due to the nature of the study, long-term follow-up was not conducted. Additionally, we only examined the microbial adhesion of *S. mutans*; other microorganisms should also be investigated in future studies to provide a more comprehensive understanding of microbial adhesion on these materials.

## Conclusions

Within the limits of this study, both milled PMMA and conventional PMMA crowns showed a reduction in microbial adhesion over time. However, milled PMMA demonstrated a significantly greater and faster decrease in *S. mutans* counts compared to conventional PMMA. While both materials eventually reduced bacterial counts, the milled PMMA crowns achieved this reduction more rapidly, indicating superior antimicrobial properties in the early post-placement period. This suggests that milled PMMA crowns may be more effective in preventing early bacterial colonization, thereby potentially reducing the risk of post-operative infections and promoting better oral health outcomes. More clinical trials are required to validate the findings of this study. 
